# Hepatoprotective Effect of* Eriobotrya japonica* Leaf Extract and Its Various Fractions against Carbon Tetra Chloride Induced Hepatotoxicity in Rats

**DOI:** 10.1155/2018/3782768

**Published:** 2018-12-16

**Authors:** Abdelaaty A. Shahat, Riaz Ullah, Ali S. Alqahtani, Mansour S. Alsaid, Husseiny A. Husseiny, Osaid T. R. Al Meanazel

**Affiliations:** ^1^Department of Pharmacognosy (Medicinal, Aromatic & Poisonous Plants Research Center (MAPPRC), College of Pharmacy, King Saud University, Riyadh 11451, Saudi Arabia; ^2^Phytochemistry Department, National Research Centre, 33 El Bohouth St., P.O. Box 12622, Dokki, Giza, Egypt; ^3^Department of Chemistry, Government College Ara Khel FR, Kohat, KPK, Pakistan; ^4^Faculty of Pharmacy, Misr University for Science and Technology (MUST), 6th of October City, Giza, Egypt; ^5^Pharmaceutical Department, College of Pharmacy, King Saud University, Riyadh, Saudi Arabia

## Abstract

*Eriobotrya japonica *is traditionally used as an antipyretic, digestive, and diuretic agent. Its flowers possess free radical–scavenging, antioxidative, and hepatoprotective effects. We investigated the hepatoprotective potential of* E. japonica *leaf extract and its various fractions against hepatotoxicity in rats. Liver injury was stimulated by the oral administration of carbon tetrachloride (CCl_4_; 2.5 mL/kg b.wt.). Male albino rats (n = 55) were distributed arbitrarily into 11 groups: Group I, normal control group; Group II, CCl_4_ (positive control group); Group III, CCl_4_ + silymarin; Groups IV and V, CCl_4_ + two doses of 250 and 500 mg/kg of the 80% methanolic extract of* E. japonica *leaves, respectively; Groups VI and VII, CCl_4_ + 250 mg/kg and 500 mg/kg of the ethyl acetate fraction, respectively; Groups VIII and IX, CCl_4_ + 250 and 500 mg/kg of the butanol fraction, respectively; and Groups X and XI, CCl_4_ + 250 and 500 mg/kg of the aqueous fraction of* E. Japonica *leaves, respectively. CCl_4_-treated rats that were given 250 or 500 mg/kg of the methanol extract of* E. Japonica *leaves, or its ethyl acetate, butanol, or aqueous fractions, had significantly lower levels of biochemical parameters such as alanine aminotransferase, aspartate transaminase, alkaline phosphate, total protein, gamma-glutamyl transferase, and bilirubin levels than those of the CCl_4 _positive group. However, the extract and fractions did not significantly affect lipid profiles. Thus, we conclude that* Eriobotrya *leaf extract and its fractions have a hepatoprotective effect against CCl_4_-induced hepatotoxicity in rats.

## 1. Introduction

Medicinal plants and their extracts represent a rich source of crude medications that possess therapeutic properties. Indeed, the World Health Organization reports that various plant fractions and their dynamic constituents are utilized as traditional medicines by 80% of the world population [1]. One such plant is* Eriobotrya japonica*, commonly known as loquat, an evergreen plant that belongs to the family Rosaceae.

*Eriobotrya japonica *originated in southeast China, but it is now grown in India, Japan, Korea, and other countries. It is small in size, with narrow leaves that are light in color on the underside and darker green on the upper surface. The leaves are valuable for the treatment of incessant maladies such as dysmenorrhea, lower back pain, asthma, chronic bronchitis, asthma, phlegm, and headache [2]. In Japan, loquat leaves have traditionally been used as antipyretic, digestive, and diuretic agents [3].

A variety of natural compounds such as tannins, megastigmane glycosides, triterpenes, sesquiterpenes, and flavonoids have been found in the leaves of* E. japonica*. Several of those compounds are active against viruses, inflammation, hyperglycemia, and tumors [2, 4–6]. Spectral methods have determined that the primary bioactive compounds of* E. japonica *leaves are tormentic acid, oleanolic acid, maslinic acid, ursolic acid, and corosolic acid [7]. Previous studies have shown that tormentic acid has normoglycemic, hypoglycemic, antiatherogenic, anti-inflammatory, and anticancer effects; and it reduces vascular smooth muscle cell expansion [8, 9].

The hydroalcoholic extracts of* E. japonica *flowers possess free radical–scavenging, antioxidative, and hepatoprotective effects against mercuric chloride induced hepatotoxicity in rats [10].

To explore the possible scavenging capacity and hepatoprotective effects of* E. japonica*, we injected male rats with carbon tetrachloride (CCl_4_), an extremely lethal chemical compound that is commonly used for inducing hepatotoxicity in animal studies [11, 12]. Then, the effects of an 80% methanol extract of* E. japonica* leaves, as well as its ethyl acetate, butanol, and aqueous fractions, against CCl_4_-induced hepatotoxicity were investigated. In this way, we aimed to scientifically validate and support the conventional uses and previous findings of* E. japonica. *

## 2. Material and Methods

### 2.1. Plant Materials and Authentication

In April 2016, the leaves of* E. japonica *were collected from Wadi Houf, Helwan, Egypt, and taxonomically confirmed by Professor Ibrahim Elgarf, Professor of Taxonomy, Faculty of Science, Cairo University, Cairo, Egypt.

### 2.2. Extraction

Dried shade leaves were powdered (1000 g) and extracted with 80% (v/v) methanol and then vacuum-concentrated under reduced pressure at 40 ± 1°C until dry. The residue was designated as methanolic extract (Er-1). A portion of Er-1 (70 g) was dissolved in water and fractionated successfully with hexane, chloroform, ethyl acetate (Er-2), and butanol (Er-3); the remaining aqueous solution was designated as Er-4.

### 2.3. Chemicals and Kits

Antioxidants, formalin, CCl_4_, isoflurane, and other chemicals were procured from El-Gomhouria Company, Cairo, Egypt. Kits used for biochemical analysis of alkaline phosphate (ALP), alanine aminotransferase (ALT), aspartate transaminase (AST), total protein, gamma-glutamyl transferase (GGT), bilirubin, cholesterol, triglycerides, high density lipoprotein (HDL), malondialdehyde (MDA), nonprotein sulfhydryl groups (NP-SH), and total protein were obtained from the Gamma Trade Company for Pharmaceutical and Chemicals, Dokki, Egypt.

### 2.4. Induction of Hepatotoxicity

Liver damage was induced by subcutaneous injections of CCl_4_ (2 mL/kg) [13].

### 2.5. Test Groups

Rats were acclimated to animal laboratory conditions at 25°C, 55% humidity, and a 12 h:12 h light-dark cycle for seven days prior to testing. Water was supplied* ad libitum*, and the rats were fed a basal diet for the entirety of the study. Afterward, 55 male rats (Laboratory Animal Colony, Helwan, Egypt) were randomly divided into 11 groups as follows. In Group I, rats were subcutaneously injected with paraffin oil and then marked as the normal control group. In Group II, rats were subcutaneously injected with CCl_4_ (2 mL/kg b.wt.) and then marked as the positive control group. In Group III, rats were subcutaneously injected with CCl_4_ (2 mL/kg b.wt.) and silymarin 10 mg/kg. Silymarin is the most used natural constituent for the healing of hepatic diseases worldwide due to its antifibrotic, anti-inflammatory, and antioxidant activities. Silymarin functions by stabilizing biological membranes and increasing protein synthesis [14]. Therefore, it is used as a standard drug around the world for hepatoprotective experiments.

In Groups IV and V, rats were subcutaneously injected with CCl_4_ (2 mL/kg b.wt.) and Er-1 (250 and 500 mg/kg, respectively). In Groups VI and VII, rats were subcutaneously injected with CCl_4_ (2 mL/kg b.wt.) and Er-2 (250 and 500 mg/kg, respectively). In Groups VIII and IX, rats were subcutaneously injected with CCl_4_ (2 mL/kg b.wt.) and Er-3 (250 and 500 mg/kg, respectively). In Groups X and XI, rats were subcutaneously injected with CCl_4_ (2 mL/kg b.wt.) and Er-4 (250 and 500 mg/kg, respectively). Experiments were performed in Biology Lab of Medicinal Aromatic and Poisonous Plants Research Center College of Pharmacy King Saud University, Saudi Arabia, April-June 2018.

At the end of four weeks, food (but not water) was withheld from all animals for 12 h. All rats were sacrificed with isoflurane. Blood samples were collected in clean centrifuge tubes via cardiac puncture. The samples were centrifuged at 3000 rpm for 15 min to separate the serum. The serum was carefully removed and transferred into lavender test tubes and solidified at 20°C until utilization for biochemical experiments.

The livers of all test rats were divided into two; one half was immediately used for biochemical analysis, and the second half was subjected to histopathology examination.

### 2.6. Biochemical Analysis

Biochemical parameters such as ALT, AST, GGT, bilirubin, and ALP were calculated with the help of Reflotron Plus analyzer and commercially available kits (Roche) [15]. Commercial diagnostic kits were used to assess lipid profiles for levels of compounds such as aggregate cholesterol, triglyceride, HDL, very low-density lipoprotein (VLDL), and low-density lipoprotein (LDL) [16]. The methods described by Ullah [15] were used to determine the serum concentrations of MDA, NP-SH, and serum total protein.

### 2.7. Histological Study

All animals were euthanized under isoflurane anesthesia and then sacrificed. Small samples of liver, kidney, and testis were immediately sliced. The sections were fixed in 10% formol saline for 24 h and then bathed in tap water, dried in increasing concentrations of ethanol, cleaned in xylene, and inserted in paraffin wax (melting point of 55–60°C). Then, 6 *μ*m thick segments were primed and stained with hematoxylin and eosin [17].

### 2.8. Statistical Analysis

Data are shown as the mean ± standard error. The level of statistical significance (P < 0.05, P < 0.01, and P < 0.001) among the groups was determined by one-way analysis of variance (ANOVA) followed by Dennett's multiple comparison test.

## 3. Results

### 3.1. Biochemical Results

Biochemical parameters of the normal control group were designated as baseline values. Serum ALP, AST, ALT, GGT, and bilirubin levels were considerably higher in the CCl_4_ group than in the control group. Those levels were considerably lower in the silymarin group than in the CCl_4_ group. Administration of 250 and 500 mg/kg of Er-1, Er-2, Er-3, and Er-4 in CCl_4_-treated rats significantly reduced the levels of ALT, AST, ALP, GGT, and bilirubin compared to those of the CCl_4_ positive control group (Table 1). Moreover, extracts and fractions+ CCl_4_-treated rats did not have significantly different cholesterol, triglyceride, LDL, HDL, and VLDL levels than did the normal control group (Table 2). Thus,* E. japonica *leaf extract and its fractions had noticeable hepatoprotective activity against CCl_4_-induced hepatotoxicity.

MDA levels indicated that lipid peroxidation was significantly higher in the CCl_4_ group than in the normal control group (P < 0.001). Pretreatment with silymarin at 10 mg/kg significantly reduced MDA to levels that nearly matched those of the control group. Although the MDA levels of CCl_4_-treated rats that were preadministered low dose (250 mg/kg) Er-1 did not significantly differ from those of the CCl_4_ positive control group, the MDA levels of CCl_4_-treated rats in preadministered high dose (500 mg/kg) of Er-1 were significantly lower than those of the CCl_4_ positive control group (P < 0.001). Postreatment of CCl_4_-treated with low- or high dose Er-2 significantly reduced MDA levels as compared to those of the CCl_4_ positive control group (P < 0.001). Similarly, treatment of CCl_4_-treated rats with low- or high dose Er-3 significantly reduced MDA levels as compared to those of the CCl_4_ positive control group (P < 0.001). Whereas rats treated with low dose Er-4 did not have significantly lower MDA levels than those in the CCl_4_ positive control group, those treated with high dose Er-4 experienced a significant reduction in MDA levels (Table 3).

In CCl_4_-treated rat's preadministered low (250 mg/kg) or high (500 mg/kg) doses of Er-1 or Er-4, there was insignificant recovery of NP-SH levels as compared to those of the CCl_4_ group. However, samples of Er-2 or Er-3 at doses of 250 or 500 mg/kg caused significant increases in NP-SH levels as compared to those of the CCl_4_ positive control group (Table 3).

Treatment of animals with CCl_4_ agents led to depletion of total protein. Pretreatment of test animals with silymarin significantly increased total protein levels as compared to those of the CCl_4_ positive control group; their total protein levels approached those of the normal control group. The total protein levels of rats that received low or high doses of Er-4 did not significantly differ from those of the CCl_4_ positive control group. In contrast, rats given low or high doses of Er-2 or Er-3 had significantly higher total protein levels than those of the CCl_4_ positive control group (Table 3).

### 3.2. Histopathological Results

**Figure 1:**
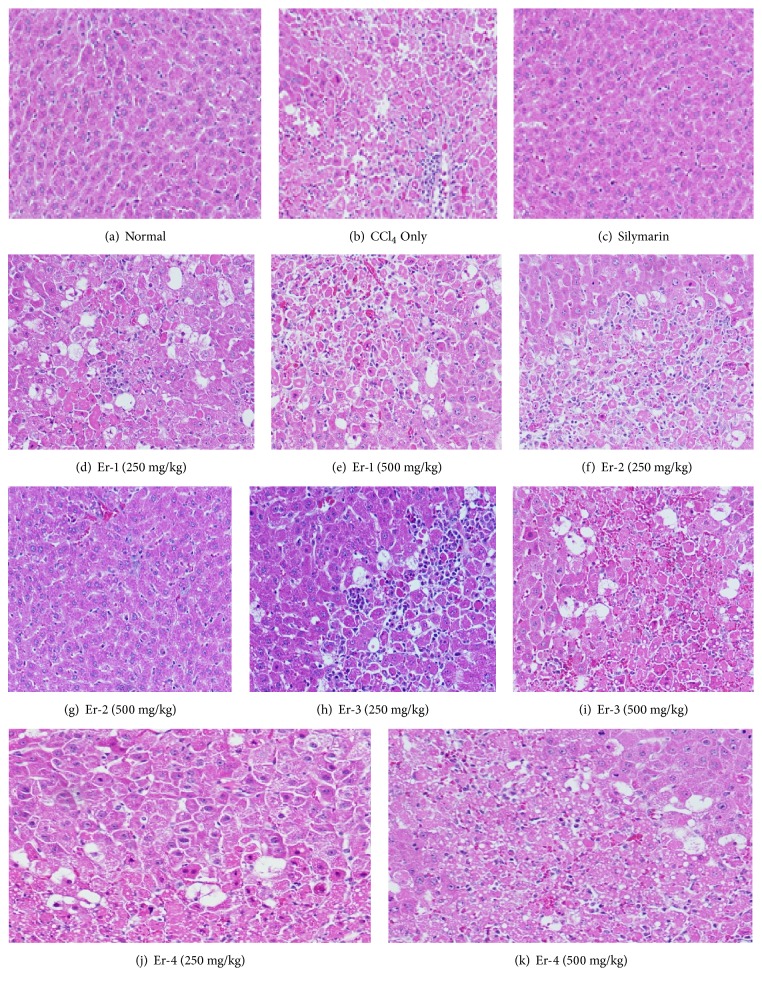
Histopathological examination of rat liver tissues (H & E stain × 150).

Microscopic histological inspection of the liver segments of normal control rats is baseline images with normal hepatocytes, and the hepatic sinusoids appeared to be lined by Kupffer cells (Figure 1(a)). In comparison, the hepatic lobules of CCl_4_ positive control rats showed severely distorted structures, congestion of the central veins and hepatic sinusoids, and inflammatory cell infiltration (Figure 1(b)). The hepatocytes of animals that were coadministered silymarin and CCl_4_ had normal architecture that resembled that of the normal control group (Figure 1(c)).

In CCl_4_-treated rats supplemented with low dose (250 mg/kg) Er-1, the hepatocytes did not completely recover; a portion of their livers had mild cellular swelling and moderate hepatocyte necrosis (Figure 1(d)). Rats treated with CCl_4_ and presupplemented with high dose (500 mg/kg) of Er-1 had normal hepatic lobules, but their hepatocytes were not normal (Figure 1(e)).

Hepatocytes did not recover to normal in CCl_4_-treated rats supplemented with low dose (250 mg/kg) of Er-2. Their liver slices revealed slight cellular swelling and moderate focal necrosis of hepatocytes (Figure 1(f)). However, CCl_4_-treated rats supplemented with high dose (500 mg/kg) of Er-2 showed normal hepatic lobules and normal hepatocytes (Figure 1(g)).

Histopathological examination of livers from CCl_4_-treated rat's preadministered low dose (250 mg/kg) of Er-3 showed abnormal hepatocytes, with mild cellular swelling and focal necrosis that is associated with inflammatory infiltration (Figure 1(h)). However, the livers of those supplemented with high dose (500 mg/kg) of Er-3 showed imprecise structural design, with vacuolated cytoplasm and cellular necrosis. Cell penetration was also detected in their hepatic lobules (Figure 1(i)).

Finally, CCl_4_-treated rats presupplemented with low dose (250 mg/kg) of Er-4 showed distorted architecture of the hepatic lobules that is associated with mild hepatocytic swelling (Figure 1(j)). In CCl_4_-treated rats that were administered high dose (500 mg/kg) of Er-4, the scale of liver injury was miniscule and hepatic lobules were normal; however, fatty changes were observed (Figure 1(k)).

## 4. Discussion

The liver is the primary organ for controlling vital functions such as digestion and detoxification of most compounds that enter the body [18, 19]. Much attention is currently focused on research involving the pharmacological applications of plant extracts and their fractions. The present study was conducted to compare the hepatoprotective effects of* E. japonica *extract and its various fractions against CCl_4_-induced hepatotoxicity in rats.

CCl_4_ is widely used to induce liver injury because its metabolism by hepatic cytochrome P450 leads to the generation of highly reactive carbon-centered trichloromethyl radicals. Such free radicals initiate lipid peroxidation chain reactions to cause liver damage [20, 21]. MDA, an end product of lipid peroxidation in the liver, is an important indicator of CCl_4_-induced toxicity in rats [22]. In the present study, high MDA levels indicated that lipid peroxidation rates were significantly greater in the CCl_4_ positive control group than in the normal control group. Preadministration of high doses of Er-1, Er-2, Er-3, or Er-4 significantly attenuated the CCl_4_-induced elevation of MDA levels.

Studies have shown that trichloromethyl radicals of CCl_4_ (the active metabolites of CCl_4_) bind covalently to macromolecules and induce peroxidative degradation of membrane lipids of endoplasmic reticuli rich in polyunsaturated fatty acids. This process leads to excessive formation and accumulation of lipids in tissues such as the liver. In addition, lipids from peripheral adipose tissue are translocated to the liver for accumulation [23, 24]. In the present study, CCl_4_ treatment reduced HDL levels and raised serum cholesterol, triglyceride, and LDL levels, which possibly reflects impairment of the liver cells' ability to metabolize lipids and transform cholesterol to bile acid for excretion.* Eriobotrya japonica *extract and its various fractions attenuated those effects.

Our results showed that CCl_4_ raised the levels of SGPT, SGOT, ALP, and bilirubin in rat serum. Such enzymes are normally present at higher levels in cell cytoplasm than in the serum. Hepatopathy causes release of AST, ALT, and bilirubin into the bloodstream in proportion to the extent of liver damage [25]. In our study, the elevation of serum AST, ALT, ALP, and total bilirubin levels in CCl_4_-treated rats indicated cellular damage and loss of cell membrane function [26]. This was confirmed by histopathological examination of their liver sections, which showed congestion of the central veins and hepatic sinusoids, inflammatory cell infiltration, and severe architectural distortion that is associated with cellular necrosis and vacuolated cytoplasm. Administration of various formulations of loquat leaves attenuated the CCl_4_-induced elevation of SGPT, SGOT, ALP, and bilirubin levels. The effects of the leaf formulations were possibly due to the presence of flavonoids.

Studies [25, 27] have reported that many herbs are an excellent source of phenolic compounds that possess antioxidant effects. Along the same vein, several studies have shown that loquat leaves have antioxidant effects [28, 29]. Natural antioxidants express their antioxidant properties by blocking lipid peroxidation via inhibition of the enzyme xanthine oxidase [29]; direct scavenging of hydroxyl, peroxyl, and superoxide radicals [30]; and inhibition of structural damage to proteins [31].

CCl_4_ causes disruption and disassociation of polyribosomes on the endoplasmic reticulum, which leads to reduction of protein biosynthesis [32]. In our study, the injection of animals with CCl_4_ caused depletion of total proteins, which indicates liver toxicity [33]. Whereas administration of Er-2 or Er-3 significantly attenuated CCl_4_-induced total protein depletion, administration of Er-1 or Er-4 did not.

The CCl_4_ positive control group had significantly lower NP-SH levels than did the normal control group. Administration of 10 mg/kg silymarin significantly reduced NP-SH levels below those of the CCl_4_ positive control group. The recovery of the NP-SH levels after treatment with silymarin was closer to that observed in the normal control group. Furthermore, whereas administration of Er-2 or Er-3 significantly attenuated the CCl_4_-induced reduction of NP-SH levels, administration of Er-1 or Er-4 did not (Table 3).

In summary, lipid peroxidation is associated with the pathogenesis of hepatic injury that is induced by the free radical reactive intermediates of CCl_4_ degradation. Lipid peroxidation is responsible for the damage to cell membranes that results in discharge of the enzymatic markers for hepatotoxicity [34]. In our study, CCl_4_ induced a marked increase in liver MDA levels that resulted in liver injury. Pretreatment with 250 or 500 mg/kg of Er-1, Er-2, Er-3, and Er-4 attenuated the CCl_4_-induced elevation of MDA enzymes, which was confirmed by the reduction in histopathological injuries.

Many researchers suggested the proposed mechanism is reducing proinflammatory mediators or increasing anti-inflammatory cytokine (such as IL-10) discharges are significant mechanisms for the anti-inflammation effects of loquat extracts [8, 13, 14, 18–21]. Such regulation was linked with restraining the activation and expression of the nuclear factor-*κ*B (NF-*κ*B) [18, 19, 22–24] and/or mitogen-activated protein kinase (MAPK) signaling pathway [15, 22, 23], which have been suggested as key regulators of the expression of inflammatory mediators in the cellular signaling pathway [4, 5, 35–39]

The relative levels of hepatoprotection afforded by the extract and fractions were as follows: Er-2 > Er-3 > Er-4 > Er-1.

## 5. Conclusion

The above results suggest that Er-1, Er-2, Er-3, and Er-4 have hepatoprotective activity and that such activity is conferred through their ability to reduce the lipid peroxidation rate, promote antioxidant effects, and inhibit CCl_4_-induced pathological changes in the liver. Er-2 and Er-3 are more effective at protecting the liver from damage than Er-1 and Er-4 and recommended for further bioscreening natural products.

## Figures and Tables

**Table 1:** Effect of various *E. Japonica *leaf extracts and fractions on serum marker enzymes in control and experimental rats.

**Parameters **	**Groups**				
	**AST** **U/L**	**ALT** **U/L**	**ALP** **U/L**	**GGT** **(U/L)**	**Bilirubin (g/dL)**
**Normal**	107.33±3.09	26.08±1.51	341.00±7.13	5.71±0.24	0.57±0.01
**CCl**_**4**_	339.16±5.28^∗∗∗**a**^	301.00±6.00^∗∗∗**a**^	586.33±7.77^∗∗∗**a**^	17.70±0.87^∗∗∗**a**^	2.94±0.06^∗∗∗**a**^
**Silymarin 10 mg/kg + CCl**_**4**_	154.66±4.95^∗∗∗**b**^	88.15±6.47^∗∗∗**b**^	396.6±711.61^∗∗∗**b**^	7.31±0.21^∗∗∗**b**^	1.00±0.04^∗∗∗**b**^
**Er-1 250 mg/kg + CCl**_**4**_	333.66±5.25^**b**^	295.83±5.24^**b**^	584.50±7.96^**b**^	15.66±0.55^**b**^	2.95±0.04^**b**^
**Er-1 500mg/kg + CCl**_**4**_	314.33±5.40^∗**b**^	291.66±5.09^**b**^	570.00±8.70^**b**^	15.23±0.35^∗**b**^	2.79±0.04^**b**^
**Er-2 250mg/kg + CCl**_**4**_	278.33±6.08^∗∗∗**b**^	196.50±6.33^∗∗∗**b**^	492.3±5.2^∗∗∗**b**^	12.71±0.35^∗∗∗**b**^	1.85±0.06^∗∗∗**b**^
**Er-2 500mg/kg + CCl**_**4**_	194.16±5.46^∗∗∗**b**^	156.66±5.32^∗∗∗**b**^	441.0±6.24^∗∗∗**b**^	10.41±0.26^∗∗∗**b**^	1.40±0.03^∗∗∗**b**^
**Er-3 250mg/kg + CCl**_**4**_	265.50±8.55^∗∗∗**b**^	170.16±7.21^∗∗∗**b**^	468.0±8.1^∗∗∗**b**^	10.11±0.24^∗∗∗**b**^	1.81±0.07^∗∗∗**b**^
**Er-3 500mg/kg + CCl**_**4**_	198.50±4.77^∗∗∗**b**^	137.16±3.09^∗∗∗**b**^	428.1±6.44^∗∗∗**b**^	8.55±0.35^∗∗∗**b**^	1.23±0.05^∗∗∗**b**^
**Er-4 250 mg/kg + CCl**_**4**_	318.50±8.00^**b**^	310.50±4.50^**b**^	594.50±7.39^**b**^	16.76±0.53^**b**^	2.98±0.04^**b**^
**Er-4 500 mg/kg + CCl**_**4**_	297.3±5.14^∗∗∗**b**^	292.33±5.00^**b**^	582.83±15.47^**b**^	16.06±0.84^**b**^	2.82±0.05^**b**^

All values represent mean ± SEM. ^∗^P < 0.05; ^∗∗^P < 0.01; ^∗∗∗^P < 0.001; ANOVA. ^**a**^As compared to that of the normal control group; ^**b**^as compared to that of the CCl_4_ positive control group.

**Table 2:** Effect of *E. Japonica *leaf extract and fractions on metabolism and serum lipoproteins in control and experimental rats.

**Parameters**	**Groups**				
	**Cholesterol (mg/dL)**	**Triglycerides (mg/dL)**	**HDL** **(mg/dL)**	**LDL** **(mg/dL)**	**VLDL** **(mg/dL)**
**Normal**	108.3±3.08	81.88±2.41	47.56±1.14	44.39±3.09	16.37±0.48
**CCl**_**4**_	231.5±8.5^∗∗∗**a**^	187.83±3.87^∗∗∗**a**^	22.93±0.95^∗∗∗**a**^	171.0±8.19^∗∗∗**a**^	37.56±0.77^∗∗∗**a**^
**Silymarin 10 mg/kg + CCl**_**4**_	128.5±3.5^∗∗∗**b**^	104.51±3.56^∗∗∗**b**^	44.6±1.30^∗∗∗**b**^	62.9±3.5^∗∗∗**b**^	20.90±0.77^∗∗∗**b**^
**Er-1 250 mg/kg + CCl**_**4**_	223.0±4.78^**b**^	192.83±4.85^**b**^	24.91±0.54^**b**^	159.5±4.3^**b**^	38.56±0.97^**b**^
**Er-1 500 mg/kg + CCl**_**4**_	211.16±4.4^**b**^	187.5±5.2^**b**^	26.43±0.39^∗∗**b**^	147.23±3.76^∗**b**^	37.50±1.04^**b**^
**Er-2 250 mg/kg + CCl**_**4**_	186.1±4.5^∗∗∗**b**^	157.5±3.7^∗∗∗**b**^	31.4±1.0^∗∗∗**b**^	123.1±4.7^∗∗∗**b**^	31.50±0.74^∗∗∗**b**^
**Er-2 500 mg/kg + CCl**_**4**_	155.5±4.1^∗∗∗**b**^	137.0±3.8^∗∗∗**b**^	38.40±0.89^∗∗∗**b**^	89.7±4.11^∗∗∗**b**^	27.4±0.77^∗∗∗**b**^
**Er-3 250 mg/kg + CCl**_**4**_	150.3±3.3^∗∗∗**b**^	128.6±3.4^∗∗∗**b**^	35.41±0.88^∗∗∗**b**^	89.1±4.19^∗∗∗**b**^	25.72±0.76^∗∗∗**b**^
**Er-3 500 mg/kg + CCl**_**4**_	129.6±8.3^∗∗∗**b**^	125.8±3.4±^∗∗∗**b**^	40.81±1.21^∗∗∗**b**^	63.6±3.8^∗∗∗**b**^	25.16±0.68^∗∗∗**b**^
**Er-4 250 mg/kg + CCl**_**4**_	219.83±7.5^**b**^	180.16±7.4^**b**^	25.58±1.13^**b**^	161.2±9.4^**b**^	36.03±1.48^**b**^
**Er-4 500 mg/kg + CCl**_**4**_	215.66±4.4^**b**^	166.33±5.16^∗**b**^	27.58±0.92^∗∗**b**^	154.8±4.8^**b**^	33.26±1.03^∗∗**b**^

All values represent mean ± SEM. ^∗^P < 0.05; ^∗∗^P < 0.01; ^∗∗∗^P < 0.001; ANOVA, followed by Dennett's multiple comparison test. ^**a**^As compared to that of the control group; ^**b**^as compared to that of the CCl_4_ positive control group.

**Table 3:** Effect of *E. japonica *leaf extract and fractions on MDA, NP-SH, and total protein levels in liver tissues.

**Treatments**	**MDA** **(nmol/g)**	**NP-SH** **(nmol/g)**	**Total Protein (g/L)**
**Normal**	1.11±0.04	6.33±0.28	121.35±3.36
**CCl**_**4**_	7.43±0.29^∗∗∗**a**^	1.41±0.21^∗∗∗**a**^	59.48±1.89^∗∗∗**a**^
**Silymarin (10 mg/kg) + CCl**_**4**_	2.15±0.09^∗∗∗**b**^	5.91±0.26^∗∗∗**b**^	106.98±2.28^∗∗∗**b**^
**Er-1 (250 mg/kg) + CCl**_**4**_	6.76±0.38^**b**^	1.77±0.29^**b**^	59.88±2.31^**b**^
**Er-1 (500 mg/kg) + CCl**_**4**_	5.59±0.24^∗∗∗**b**^	2.20±0.25^∗**b**^	66.66±1.68^∗**b**^
**Er-2 (250 mg/kg) + CCl**_**4**_	4.25±0.18^∗∗∗**b**^	4.81±0.21^∗∗∗**b**^	73.45±1.00^∗∗∗**b**^
**Er-2 (500 mg/kg) + CCl**_**4**_	3.60±0.12^∗∗∗**b**^	5.13±0.28^∗∗∗**b**^	81.83±1.89^∗∗∗**b**^
**Er-3 (250 mg/kg) + CCl**_**4**_	3.60±0.19^∗∗∗**b**^	5.31±0.13^∗∗∗**b**^	77.44±2.59^∗∗∗**b**^
**Er-3 (500 mg/kg) + CCl**_**4**_	2.79±0.10^∗∗∗**b**^	5.00±0.27^∗∗∗**b**^	95.80±2.83^∗∗∗**b**^
**Er-4 (250 mg/kg) + CCl**_**4**_	6.87±0.3^**b**^	1.52±0.2^**b**^	58.68±1.60^**b**^
**Er-4 (500 mg/kg) + CCl**_**4**_	5.52±0.21^∗∗∗**b**^	2.50±0.28^∗**b**^	67.46±1.43^∗∗**b**^

All values represent mean ± SEM. ^∗^P < 0.05; ^∗∗^P < 0.01; ^∗∗∗^P < 0.001; ANOVA, followed by Dennett's multiple comparison test. ^**a**^As compared to that of the control group; ^**b**^as compared to that of the CCl_4_ positive control group.

## Data Availability

All data obtained during this study are included in this article. The data or any material can also be obtained from corresponding author upon reasonable request.
